# Supplementary material for: Age-Specific Effects of Visual Feature Binding
Source: Brain Sci. 2023 Sep 29;13(10):1389. doi: 10.3390/brainsci13101389 (PMC10605270; doi:10.3390/brainsci13101389)
Supplement: Supplementary file 1 [file brainsci-13-01389-s001.zip › brainsci-2623876-supplementary.pdf]

Supplement: Overview

Content	Number
Neuropsychological Test Scores in old participants	Table S1
Grand-averages: ERP responses to test arrays	Figure S1
Peak analysis: P1 and N2	Analysis S1
Effects of the factors block	Analysis S2
Statistical analysis of hit rate and false alarms	Table S2
Comparison of studies: Age-specific binding effect	Table S3

Supplement: Table S1

Neuropsychological Test Scores: Comparison of the non-standardized test values

Test	OLD	YOUNG	t-Test
Trail-Making-Test (A)	35.3 s (2.16)	24.5 s (1.44)	-4.24 ***
Trail-Making-Test (B)	81.30 s (30.52)	51.3 s (17.53)	-4.21 ***
Block Span (forward)	7.56 (1.68)	9.73 (1.89)	4.11 ***
Block Span (backward)	8.00 (1.57)	9.54 (1.50)	3.46 ***
IDG: visual learning	5.00 (2.37)	8.92 (1.23)	7.36 ***
IGD: visual recognition	7.32 (1.49)	9.77 (0.51)	7.85 ***

IGD: visual association	4.82 (2.04)	6.92 (0.39)	5.16 ***
LPS: reasoning	19.45 (5.74)	31.96 (4.07)	8.80 ***
LPS: visual filtering	21.91 (5.30)	29.19 (3.90)	5.48 ***

*** $p<0.001$, ** $p<0.01$, * $p<0.05$

IGD (Inventar zur Gedächtnisdiagnostik, Baller, Brand, Kalbe & Kessler, 2006, Hogrefe Testzentrale) LPS (Leistungsprüfsystem, Horn, 1983, Hogrefe Testzentrale)

Supplement: Figure S1

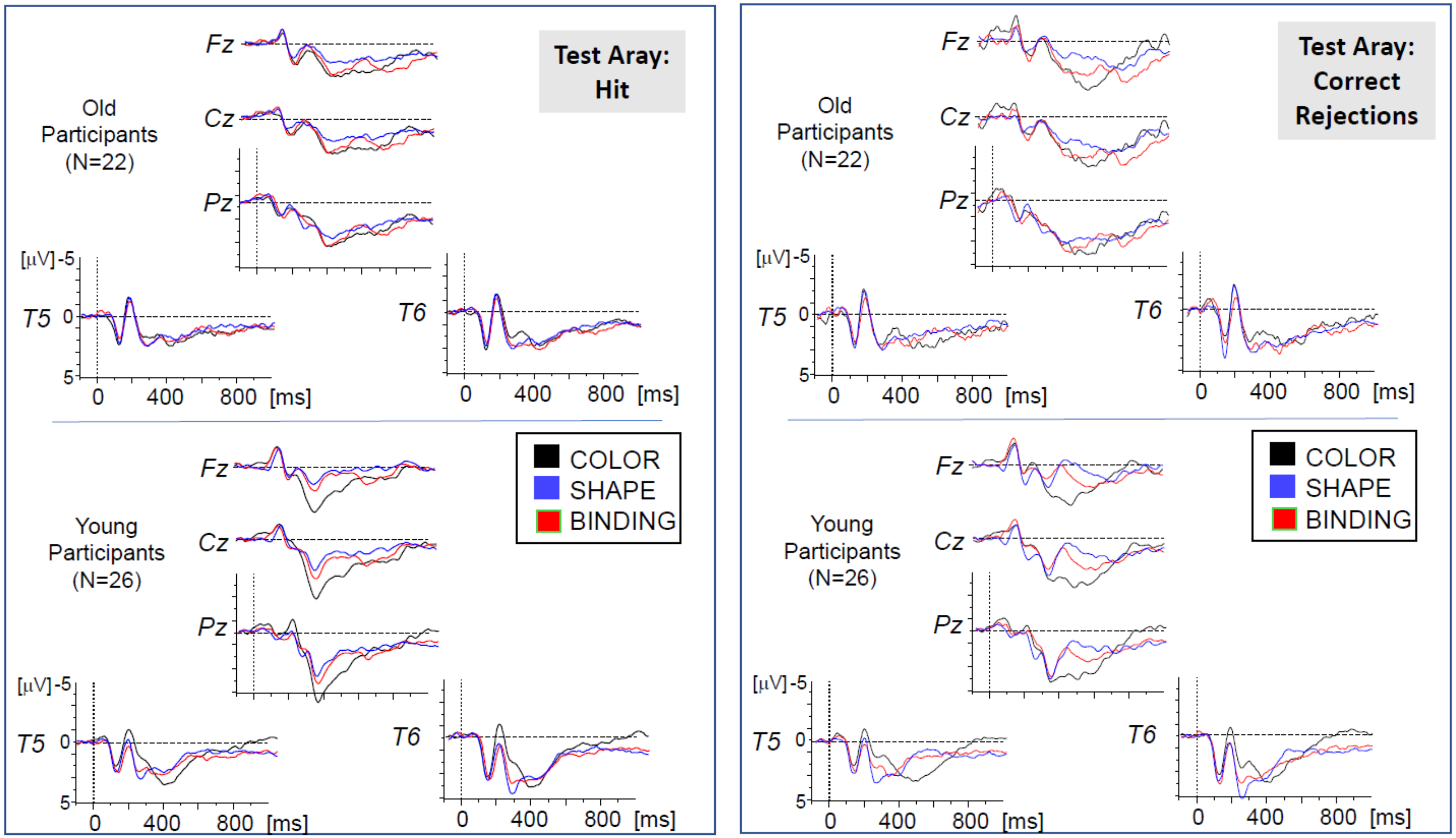

Supplement: Analyis S1

Peak analysis: N1 and P2

Coding of Condition

1=Color, 2=Shape, 3=Binding

N1 latency

Group	Condition	Mean	SD
young	1	187,673	1,969
	2	182,173	1,866
	3	183,712	1,943
old	1	191,545	2,140
	2	188,432	2,028
	3	190,545	2,112

N1 amplitude

Group	Condition	Mean	SD
young	1	-3,272	,503
	2	-3,734	,478
	3	-2,938	,471
old	1	-3,711	,547

	2	-4,414	,520
	3	-3,614	,512

P2 latency

Group	Condition	Mean	SD
young	1	235,615	2,119
	2	237,519	2,248
	3	234,788	2,398
old	1	240,045	2,304
	2	243,295	2,444
	3	241,500	2,607

P2 amplitude

Group	Condition	Mean	SD
young	1	5,767	,498
	2	4,302	,532
	3	5,015	,488
old	1	2,324	,541
	2	2,086	,578
	3	2,571	,530

Results of the ANOVA (effects of 'condition' and 'condition x age') and subsequent post-hoc comparisons for the behavioral data (hit rate, A'). (B = Binding, C = feature-alone color, S = feature-alone shape).

Variable		Contrast	Factor Condition	Interaction Condition x Age
**N1 (Latency)**			$F(2,92)=7.54$, $p=.001$, $\eta_p^2=.141$	$F(2,92)=.99$, $p=.374$, $\eta_p^2=.021$
	post-hoc	B vs C	$F(1,46)=4.26$, $p=.045$, $\eta_p^2=.085$	n.a.
		B vs S	$F(1,46)=3.52$, $p=.067$, $\eta_p^2=.067$	n.a.
		C vs S	$F(1,46)=14.72$, $p<.001$, $\eta_p^2=.239$	n.a.
**N1 (Amplitude)**			$F(2,92)=9.50$, $p<.001$, $\eta_p^2=.171$	$F(2,92)=0.26$, $p=.768$, $\eta_p^2=.006$
	post-hoc	B vs C	$F(1,46)=1.43$, $p=.238$, $\eta_p^2=.030$	n.a.
		B vs S	$F(1,46)=14.46$, $p<.001$, $\eta_p^2=.239$	n.a.
		C vs S	$F(1,46)=10.74$, $p=.002$, $\eta_p^2=.186$	n.a.
**P2 (Latency)**			$F(2,92)=1.60$, $p=.208$, $\eta_p^2=.034$	$F(2,92)=.27$, $p=.77$, $\eta_p^2=.006$
	post-hoc	B vs C	n.a.	n.a.
		B vs S	n.a.	n.a.
		C vs S	n.a.	n.a.

**P2 (Amplitude)**		$F(2,92)=5.86$, $p=.004$, $\eta_p^2=.113$	$F(2,92)=3.26$, $p=.043$, $\eta_p^2=.066$
post-hoc	B vs C	$F(1,46)=1.07$, $p=.306$, $\eta_p^2=.023$	old: $F(1,21)=.41$, $p=.530$, $\eta_p^2=.019$<br>young $F(1,25)=6.03$, $p=.021$, $\eta_p^2=.194$
	B vs S	$F(1,46)=4.92$, $p=.031$, $\eta_p^2=.097$	old: $F(1,21)=2.46$, $p=.132$, $\eta_p^2=.105$<br>young: $F(1,25)=2.86$, $p=.103$, $\eta_p^2=.103$
	C vs S	$F(1,46)=11.68$, $p=.001$, $\eta_p^2=.199$	old: $F(1,21)=.62$, $p=.440$, $\eta_p^2=.029$<br>young: $F(1,25)=14.36$, $p=.001$, $\eta_p^2=.365$

Supplement: Analyis S2

Statistical effects of the experimental factors age and block, and their interaction effect with the factor condition.

Variable: Discrimination Ability (A')

Group	*Block*	*Task*	*Mean*	*SD*
young	*1*	*Color*	*,985*	*,013*
	2	*Color*	*,987*	*,004*
	1	*Shape*	*,954*	*,016*
	2	***Shape***	***,983***	***,007***
	2	*Binding*	*,923*	*,020*
	2	*Binding*	*,949*	*,017*
old	*1*	*Color*	*,961*	*,014*
	2	*Color*	*,990*	*,004*
	1	*Shape*	*,863*	*,017*
	2	*Shape*	*,948*	*,008*
	2	*Binding*	*,739*	*,023*
	2	*Binding*	*,830*	*,019*

Variable: Mean N1 activity

Gruppe	Condition	Block	Mean	SD
young	Color	1	-2,280	,487
		2	-2,234	,489
	Shape	1	-2,664	,532
		2	-2,446	,4575
	Binding	1	-2,044	,446
		2	-1,612	,436
old	Color	1	-2,741	,529
		2	-2,512	,531
	Shape	1	-3,613	,578
		2	-3,142	,497
	Binding	1	-2,511	,485
		2	-2,549	,474

Variable: Mean P2 activity

Gruppe	Condition	Block	Mean	SD
young	Color	1	4,945	,552
		2	5,067	,478
	Shape	1	2,991	,585
		2	3,771	,558
	Binding	1	3,785	,593
		2	4,169	,495
old	Color	1	,927	,600
		2	1,478	,520
	Shape	1	,751	,636
		2	1,242	,607
	Binding	1	1,035	,645
		2	1,877	,538

Variable: Mean LPC activity

Gruppe	Condition	Block	Mean	SD
young	Color	1	2,741	,467
		2	2,455	,416
	Shape	1	2,951	,498
		2	3,029	,390
	Binding	1	3,272	,459
		2	3,324	,423
old	Color	1	1,305	,508
		2	2,040	,452
	Shape	1	,592	,542
		2	1,406	,424
	Binding	1	2,048	,499
		2	2,825	,460

Results of the ANOVA (F-Statistics=

Variable	Factor age	Factor Block	Interaction Block x Condition x Age
**A'**	$F(1,46)=27.00, p<.001, \eta_p^2=.375$	$F(1,46)=48.90, p<.001, \eta_p^2=.521$	$F(2,92)=1.14, p=.325, \eta_p^2=.025$
N1	$F(1,46)=1.006, p=.321, \eta_p^2=.021$	$F(1,46)=3.01, p=.086, \eta_p^2=.063$	$F(2,92)=0.95, p=.392, \eta_p^2=.020$
P2	$F(1,46)=18.41, p<.001, \eta_p^2=.286$	$F(1,46)=7.78, p=.008, \eta_p^2=.145$	$F(2,92)=0.44, p=.640, \eta_p^2=.010$
LPC	$F(1,46)=5.50, p=.023, \eta_p^2=.107$	$F(1,46)=4.72, p=.035, \eta_p^2=.093$	$F(2,92)=0.09, p=.911, \eta_p^2=.002$

Supplement: Table S2

Results of the ANOVA (effects of 'condition' and 'condition x age') and subsequent post-hoc comparisons for the behavioral data (hit rate, false alarms). (B = Binding, C = feature-alone color, S = feature-alone shape).

Variable		Contrast	Factor Condition	Interaction Condition x Age
**Hit Rate**			*F(2,92)=12.00, p<.001,* η_p^2*=.207*	*F(2,92)=4.70, p=.012,* η_p^2*=.093*
	post-hoc	B vs C	*F(1,46)=21.58, p<.001,* η_p^2*=.319*	*Old: F(1,21)=17.66, p<.001,* η_p^2*=.457*<br>Young F(1,25)=2.40, p=.341, η_p^2=.087
		B vs S	F(1,46)=.66, p=.664, η_p^2=.004	Old: F(1,21)=0.55, p=.465, η_p^2=.026<br>Young F(1,25)=.65, p=.654, η_p^2=.026
**False Alarms**			*F(2,92)=138.94, p<.001,* η_p^2*=.757*	*F(2,92)=27.71, p<.001,* η_p^2*=.376*
post-hoc		B vs C	*F(1,46)=194.91, p<.001,* η_p^2*=.809*	*Old: F(1,21)=128.39, p<.001,* η_p^2*=.859*<br>Young F(1,25)=53.17, p<.001, η_p^2=.680
		B vs S	F(1,46)=101.47, p<.001, η_p^2=.688	Old: F(1,21)=61.15, p<.001, η_p^2=.744<br>Young F(1,25)=34.69, p<.001, η_p^2=.581

Supplement: Table S3

Comparision of experimental setups used in studies on age-specific binding capacity

Study<br>Reference: see Text	sample (young)	sample (old)	Type: task
**Niedeggen et al.**	N=26 (mean age 23.8)	N=22 (mean age 68.8)	change detection
**Brockmole et al., (2008)**			
Experiment 2	N=12 (mean age 21.5)	N=12 (mean age 67.3)	change detection
**Isella et al., 2015**	N=25 (mean age 24.8)	N=26 (mean age 73.2)	change detection
**Parra et al., 2009**	N=14 (mean age 20.7)	N=14 (mean age 65.9)	change detection
**Pertzow et al., 2015**	N=32 (mean age 25.2) and<br>N=53 (mean age 35.2)	N=35 (mean age 49.2) and<br>N=35 (mean age 67.5)	item recognition
**Brown & Brockmole, 2010**			
Experiment 1	N=24 (mean age 18.9)	N=24 (mean age 72.2)	single item recognition
Experiment 2	N=24 (mean age 20.3)	N=24 (mean age 72.3)	single item recognition
**Chalfonte & Johnson, 1996**			
Experiment 1b	N=32 (mean age 19.1)	N=32 (mean age 69.5)	item recognition
Experiment 2	N=32 (mean age 19.4)	N=32 (mean age 71.0)	item recognition
Experiment 3b	N=32 (mean age 19.9)	N=32 (mean age 71.9)	item recognition
**Cowan et al., 2006**			
Experiment 1a	N=53 (mean age 20.4)	N=33 (mean age 71.1)	change detection
Experiment 2a	N=24 (mean age 18.9)	N=32 (mean age 71.4)	change detection
**Mitchel et al., 2000**			
Experiment 1	N=24 (mean age 19.5)	N=24 (mean age 74.1)	change detection
Experiment 2	N=16 (mean age 19.6)	N=16 (mean age 75.1)	change detection
**Rhodes et al., 2017**<br>*Experiment 1*	N=24 (mean age 20.7) and<br>N= 24 (mean age 21.1)	N=24 (mean age 70.9)<br>and N=25 (mean age 70.2)	change detection
Experiment 2	N=24 (mean age 20.7) and<br>N= 24 (mean age 21.1)	N=25 (mean age 70.0) and<br>N=24 (mean age 71.4)	change detection
**Brown et al., 2016**			
Experiment 1	N=24 (mean age 19.9)	N=24 (mean age76.0)	single item recognition
Experiment 2	N=24 (mean age 22.1)	N=24 (mean age 77.6)	single item recognition
Experiment 3	N=24 (mean age 20.5)	N=24 (mean age 75.4)	single item recognition
**van Geldorb et al., 2014**	N=31 (mean age 22.3)	N= 30 (mean age 54.8 and<br>N=30 (mean age 70.2)	item recall

Supplement 6

Comparision of experimental setups used in studies on age-specific binding capacity

Study<br>Reference: see Text	Type: Feature binding	Number of Items	Presentation time: study array	Maintenance time
**Niedeggen et al.**	intrinsic intra-item (color/shape)	3	2000 ms	900 ms
**Brockmole et al., (2008)**				
Experiment 2	intrinsic intra-item (color/shape)	4	1000 ms	900 ms
**Isella et al., 2015**	intrinsic intra-item (color/shape)	4	4000ms	1000ms
**Parra et al., 2009**	intrinsic intra-item (color/color)	3 and 4	2000ms and 1000ms	900ms
**Pertzow et al., 2015**	extrinsic intra-item (object/location)	1 and 3	1000ms and 3000ms	1000ms and 4000ms
**Brown & Brockmole, 2010**				
Experiment 1	intrinsic intra-item (color/shape)	3	900ms	1000ms
Experiment 2	intrinsic intra-item (color/shape)	3	1500ms	1000ms
**Chalfonte & Johnson, 1996**				
Experiment 1b	intrinsic (color/object) and extrinsic (object/location)	30	90sec	immediate
Experiment 2	intrinsic (color/object)	30	90sec	immediate
Experiment 3b	intrinsic (color/object)	30	90sec	immediate
**Cowan et al., 2006**				
Experiment 1a	extrinsic intra-item (object/location)	4-10	250ms	immediate
Experiment 2a	extrinsic intra-item (object/location)	4-10	250ms	immediate
**Mitchel et al., 2000**				
Experiment 1	extrinsic intra-item (object/location)	9	1000ms per Item	8000ms
Experiment 2	extrinsic intra-item (object/location)	9	1000ms per Item	8000ms
**Rhodes et al., 2017**				
Experiment 1	intrinsic (color/shape)	9	900ms	1000ms
Experiment 2	extrinsic intra-item (object/location)	9	900ms	1000ms
**Brown et al., 2016**				
Experiment 1	intrinsic intra-item (color/shape)	3	900ms and 1500ms	1000ms
Experiment 2	intrinsic intra-item (color/shape)	3	1500ms	1000ms
Experiment 3	intrinsic intra-item (color/shape)	3	900ms	1000ms
**van Geldorb et al., 2014**	intrinsic intra-item (color/shape) and relational (color/shape)	4	4000ms	1000ms

Supplement 6

Comparision of experimental setups used in studies on age-specific binding capacity

**Study**	**Performance: variable**	**Contrasts**	**Age-specific effect**
Reference: see Text	A' and d' refer to discrimination ability	Comparison of experimental conditions reported in the study	
**Niedeggen et al.**	A' and hit rate	Binding<Color and Binding<Shape	yes
**Brockmole et al., (2008)**			
Experiment 2	not specified (A' or proportion correct)	Binding=Shape<Color	no
**Isella et al., 2015**	A'	Binding<Color; Color=Shape; Shape=Binding	no
**Parra et al., 2009**	A'	Binding=Non-Conjunctive Colors<Colors and Binding<Non-Conjunctive Colors<Colors	no
**Pertzow et al., 2015**	swap errors	Between Age Groups	no
**Brown & Brockmole, 2010**			
Experiment 1	A'	Shape<Color; Binding=Shape	no
Experiment 2	A'	Binding<Shape<Color	yes
**Chalfonte & Johnson, 1996**			
Experiment 1b	A'	Intrinsic vs. Extrinsic Binding	yes
Experiment 2	A'	Binding<Item and Binding<Color	yes
Experiment 3b	A'	Incidental vs. Intentional Learning of Bound Objects	yes
**Cowan et al., 2006**			yes
Experiment 1a	d'	Binding Change<Item Change	yes
Experiment 2a	d'	Binding Change<Item change	yes
**Mitchel et al., 2000**			
Experiment 1	d'	Binding<Single Feature (Object/Location)	yes
Experiment 2	d'	Binding<Single Feature (Object/Location)	yes
**Rhodes et al., 2017**			
Experiment 1	Corrected recognition P_r	Binding<Color and Binding=Shape	no
Experiment 2	Corrected recognition P_r	Binding=Color=Location	no
**Brown et al., 2016**			
Experiment 1	A'	Binding=Shape<Color (different retention times)	no
Experiment 2	A'	Binding=Shape<Color (sequential presentation)	no
Experiment 3	A'	Binding<Shape<Color (suffix interference)	yes
**van Geldorb et al., 2014**	number correct	relational<conjunctional binding<br>interference<non-interference (stronger in relational binding)	yes
